# Editorial: Navigating the landscape of *IDH*-mutant gliomas: advances in diagnosis, therapeutic management, and long-term monitoring

**DOI:** 10.3389/fonc.2026.1926822

**Published:** 2026-07-21

**Authors:** Alessia Pellerino, Giulia Berzero, Francesco Bruno

**Affiliations:** 1 Division of Neuro-Oncology, Department of Neuroscience “Rita Levi Montalcini”, University and City of Health and Science Hospital, University of Turin, Turin, Italy; 2 Neurosurgical Oncology Unit, IRCCS Ospedale Galeazzi Sant’Ambrogio, Milan, Italy

**Keywords:** IDH mutation, lower-grade gliomas, multidisciplinary management, noninvasive diagnosis, prognostic prediction, therapeutic targeting, treatment decisions

The aim of the Research Topic *“Navigating the Landscape of *IDH*-Mutant Gliomas: Advances in Diagnosis, Therapeutic Management, and Long-Term Monitoring”* is to provide a comprehensive overview of the current state of *IDH*-mutant glioma research, spanning diagnostic challenges, emerging treatment strategies, and long-term patient care. In this editorial, we will present the report from five manuscripts included in this Research Topic (four original research articles, and one perspective).

Integrated diagnosis, incorporating both molecular markers and histology (1), has reshaped the classification and prognostic stratification of lower-grade gliomas (2). The 2021 WHO Classification has identified three biologically and clinically distinct entities based on the isocitrate dehydrogenase (*IDH*) status and the chromosome 1p/19q codeletion: *IDH*-mutant 1p/19q codeleted (oligodendrogliomas), *IDH*-mutant 1p/19q intact (astrocytomas), and *IDH*-wildtype gliomas (1, 3).

As early drivers of gliomagenesis (4), *IDH1/2* mutations are associated with improved survival (5, 6) and a better response to alkylating agents (6). Therefore, their presence significantly influences patient management. The recent advent of IDH inhibitors (7) has further emphasized the clinical relevance of *IDH1/2* mutations, and the need for non-invasive methods of assessment. Liquid biopsies of cerebrospinal fluid (8) or plasma (9), magnetic resonance spectroscopy (10, 11), and radiomics (12) are currently under investigation as non-invasive approaches for the determination of the *IDH* status. However, resources, time, and expertise limit the widespread implementation of those methods into routine clinical practice. Relying on minimal human input and supervision, deep learning frameworks are emerging and accessible tools for extracting molecular information from neuroimaging. Xie et al. reported a weakly supervised deep learning method able to predict the *IDH* status from preoperative magnetic resonance imaging with good accuracy, reduced annotation time, and limited manual effort, thus warranting further investigation in molecularly annotated prospective cohorts.

Despite the prognostic relevance of molecular markers, their integration with historical criteria into a unified consensus remains incomplete. Developed in the early 2000s based on clinical trials started over a decade earlier, the prognostic criteria of the European Organization for Research and Treatment of Cancer (EORTC) (13) and the Radiation Therapy Oncology Group (RTOG) (14) do not include molecular markers (e.g., *CDKN2A/B* homozygous deletion) nor account for the recent evidence generated in the molecular era, which has attenuated or even challenged (15) the prognostic value of some traditional factors (**Figure 1**).

**Figure 1:**
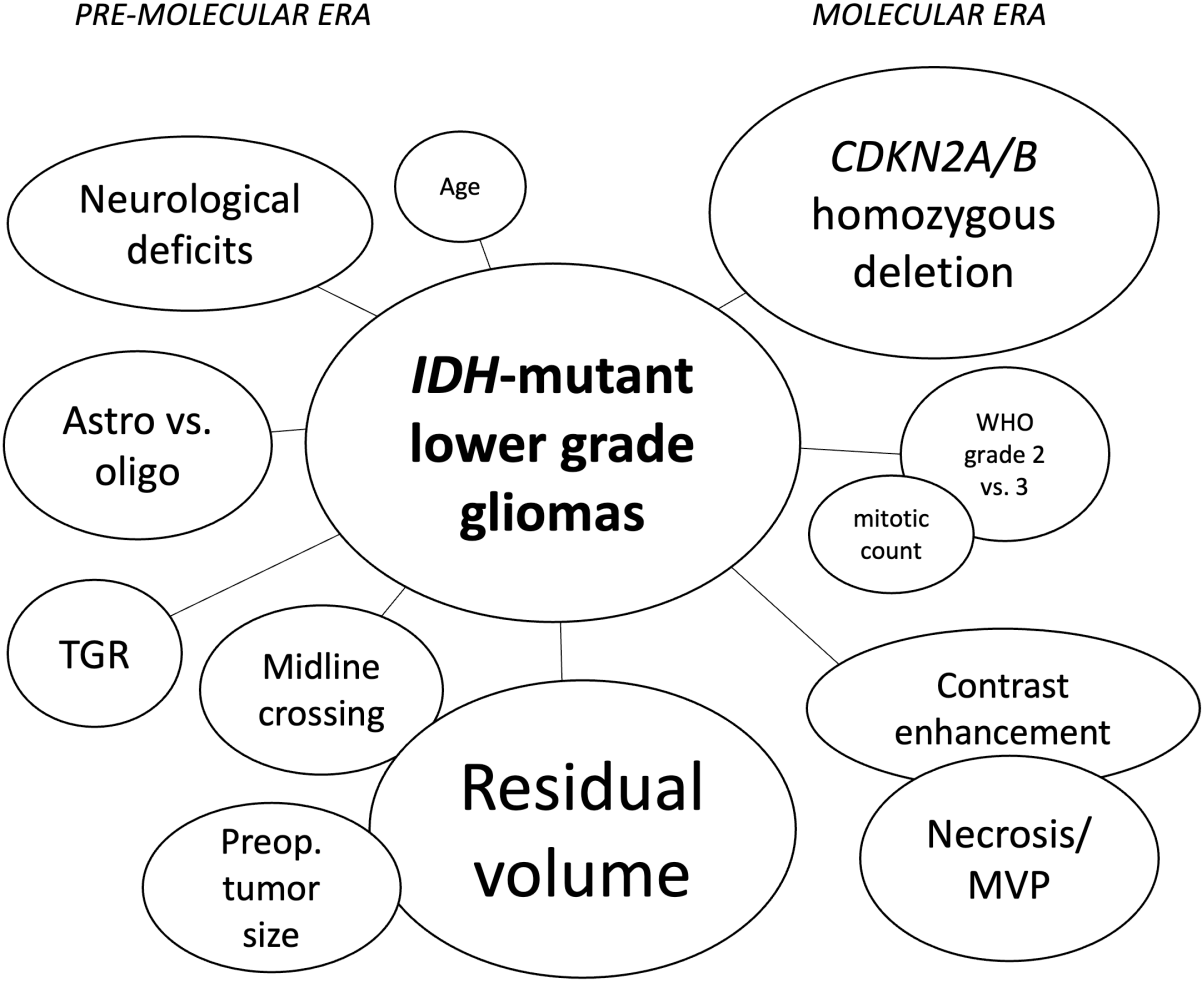
An illustrative scheme reporting the relative importance of prognostic risk factors in *IDH*-mutant lower-grade gliomas navigating towards the molecular era. MVP = microvascular proliferation; Preop., preoperative; TGR, tumor growth rate; WHO, World Health Organization.

In a large study on 253 adults with lower-grade gliomas, Carstam et al. showed that histological grade (WHO grade 2 vs. 3) did not significantly impact prognosis in *IDH*-mutant *CDKN2A/B*-intact tumors, either when analyzed collectively or when astrocytomas and oligodendrogliomas were considered separately. Similarly, age did not significantly impact survival in multivariable analysis, despite the historical prognostic cut-off of 40 years in low-grade gliomas (13). Conversely, residual tumor volume—particularly in astrocytomas (16)—was correlated with a worse outcome, as well as the presence of *CDKN2A/B* homozygous deletions. Similar findings were reported by Aprile et al., who analyzed a cohort of 61 patients with *IDH*-mutant grade 2 gliomas who underwent active observation after surgery: residual tumor diameters and midline crossing, but not age, were predictive of a shorter progression-free survival.

These observations are consistent with other reports (15) underscoring the need to reassess traditional prognostic indicators that have lost robustness in the molecular era, to incorporate novel genetic markers, and to establish standardized criteria for measuring residual tumor volume, which has recently been confirmed as a key prognostic factor in *IDH*-mutant gliomas (17). Updated and refined prognostic criteria are crucial to tailor treatment decisions, which may range from watch-and-wait or IDH inhibition in “low-risk” patients to sequential radio-chemotherapy in “high-risk” patients (18–20).

Given the relatively prolonged disease course of *IDH*-mutant gliomas, their management is highly challenging and requires to carefully balance the achievement of long-lasting tumor control and preservation of neurological function. This balance begins with surgical resection itself: achieving maximal safe resection while preserving neurological function is difficult, and knowledge of white matter connectivity is key to this goal. In a study of 51 glioma patients, Salvati et al. showed that preoperative tractography (DTI-MRI) can help identify involvement of the frontal aslant tract (FAT), reducing the risk of verbal fluency disorders from its intraoperative damage. Preserving neurological function matters beyond surgery as well: uncontrolled seizures or complex antiseizure polytherapy may negatively affect neurological function status and impair the ability to drive or maintain independence in daily life activities. Additionally, neurocognitive deficits related to radiotherapy can heavily impact work ability, family, and social life, often supporting radiation deferral. To achieve an optimal balance between tumor control and quality of life, a multidisciplinary management is generally recommended. Aucoin et al. reported a multidisciplinary care model that includes neurosurgeons, neuro-oncologists, and epileptologists, who jointly evaluate patients after a collective review of imaging and clinical data. A neuropsychologist, a social worker, and a nurse coordinator are available on demand to address relevant practical and psychological needs in selected cases. Although quantitative evidence supporting the benefits of a multidisciplinary model are still limited, patients with *IDH*-mutant gliomas are likely to derive benefit from this approach, given the complexity of their disease (21).

In conclusion, the landscape of *IDH*-mutant gliomas is rapidly evolving, driven by advances in molecular biology, imaging, and therapeutic innovation. These progresses highlight critical gaps in prognostic modeling and clinical integration that require coordinated efforts. The definition of risk stratification, as well as the validation of emerging technologies, and the implementation of multidisciplinary care will be crucial to fully translate scientific advances into meaningful improvements in patient outcomes.

## References

[1] Louis DN Perry A Reifenberger G von Deimling A Figarella-Branger D Cavenee WK . The 2016 World Health Organization classification of tumors of the central nervous system: a summary. Acta Neuropathol. (2016) 131:803–20. doi: 10.1007/s00401-016-1545-1 27157931

[2] Tabouret E Nguyen AT Dehais C Carpentier C Ducray F Idbaih A . Prognostic impact of the 2016 WHO classification of diffuse gliomas in the French POLA cohort. Acta Neuropathol. (2016) 132:625–34. doi: 10.1007/s00401-016-1611-8 27573687

[3] Louis DN Perry A Wesseling P Brat DJ Cree IA Figarella-Branger D . The 2021 WHO classification of tumors of the central nervous system: a summary. Neuro Oncol. (2021) 23:1231–51. doi: 10.1093/neuonc/noab106 34185076 PMC8328013

[4] Watanabe T Nobusawa S Kleihues P Ohgaki H . IDH1 mutations are early events in the development of astrocytomas and oligodendrogliomas. Am J Pathol. (2009) 174:1149–53. doi: 10.2353/ajpath.2009.080958 19246647 PMC2671348

[5] Sanson M Marie Y Paris S Idbaih A Laffaire J Ducray F . Isocitrate dehydrogenase 1 codon 132 mutation is an important prognostic biomarker in gliomas. J Clin Oncol. (2009) 27:4150–4. doi: 10.1200/JCO.2009.21.9832 19636000

[6] Houillier C Wang X Kaloshi G Mokhtari K Guillevin R Laffaire J . IDH1 or IDH2 mutations predict longer survival and response to temozolomide in low-grade gliomas. Neurology. (2010) 75:1560–6. doi: 10.1212/WNL.0b013e3181f96282 20975057

[7] Rudà R Horbinski C van den Bent M Preusser M Soffietti R . IDH inhibition in gliomas: from preclinical models to clinical trials. Nat Rev Neurol. (2024) 20:395–407. doi: 10.1038/s41582-024-00967-7 38760442

[8] Miller AM Shah RH Pentsova EI Pourmaleki M Briggs S Distefano N . Tracking tumour evolution in glioma through liquid biopsies of cerebrospinal fluid. Nature. (2019) 565:654–8. doi: 10.1038/s41586-019-0882-3 30675060 PMC6457907

[9] Boisselier B Gállego Pérez-Larraya J Rossetto M Labussière M Ciccarino P Marie Y . Detection of IDH1 mutation in the plasma of patients with glioma. Neurology. (2012) 79:1693–8. doi: 10.1212/WNL.0b013e31826e9b0a 23035067

[10] Choi C Raisanen JM Ganji SK Zhang S McNeil SS An Z . Prospective longitudinal analysis of 2-hydroxyglutarate magnetic resonance spectroscopy identifies broad clinical utility for the management of patients with IDH-mutant glioma. J Clin Oncol. (2016) 34:4030–9. doi: 10.1200/JCO.2016.67.1222 28248126 PMC5477829

[11] Di Stefano AL Nichelli L Berzero G Valabregue R Touat M Capelle L . *In vivo* 2-hydroxyglutarate monitoring with edited MR spectroscopy for the follow-up of IDH-mutant diffuse gliomas: the IDASPE prospective study. Neurology. (2023) 100:e94–e106. doi: 10.1212/WNL.0000000000201137 36180241 PMC9827125

[12] Bhandari AP Liong R Koppen J Murthy SV Lasocki A . Noninvasive determination of IDH and 1p19q status of lower-grade gliomas using MRI radiomics: a systematic review. AJNR Am J Neuroradiol. (2021) 42:94–101. doi: 10.3174/ajnr.A6875 33243896 PMC7814803

[13] Pignatti F van den Bent M Curran D Debruyne C Sylvester R Therasse P . Prognostic factors for survival in adult patients with cerebral low-grade glioma. J Clin Oncol. (2002) 20:2076–84. doi: 10.1200/JCO.2002.08.121 11956268

[14] Shaw EG Berkey B Coons SW Bullard D Brachman D Buckner JC . Recurrence following neurosurgeon-determined gross-total resection of adult supratentorial low-grade glioma: results of a prospective clinical trial. J Neurosurg. (2008) 109:835–41. doi: 10.3171/JNS/2008/109/11/0835 18976072 PMC3833272

[15] van den Bent MJ French PJ Brat D Tonn JC Touat M Ellingson BM . The biological significance of tumor grade, age, enhancement, and extent of resection in IDH-mutant gliomas: How should they inform treatment decisions in the era of IDH inhibitors? Neuro Oncol. (2024) 26:1805–22. doi: 10.1093/neuonc/noae107 38912846 PMC11449017

[16] Wijnenga MMJ French PJ Dubbink HJ Dinjens WNM Atmodimedjo PN Kros JM . The impact of surgery in molecularly defined low-grade glioma: an integrated clinical, radiological, and molecular analysis. Neuro Oncol. (2018) 20:103–12. doi: 10.1093/neuonc/nox176 29016833 PMC5761503

[17] Karschnia P Young JS Wijnenga MMJ Sciortino T Teske N Corell A . A prognostic classification system for extent of resection in IDH-mutant grade 2 glioma: an international, multicentre, retrospective cohort study with external validation by the RANO resect group. Lancet Oncol. (2025) 26:1638–50. doi: 10.1016/S1470-2045(25)00534-0 41308678

[18] Weller M van den Bent M Preusser M Le Rhun E Tonn JC Minniti G . EANO guidelines on the diagnosis and treatment of diffuse gliomas of adulthood. Nat Rev Clin Oncol. (2021) 18:170–86. doi: 10.1038/s41571-020-00447-z 33293629 PMC7904519

[19] Miller JJ Gonzalez Castro LN McBrayer S Weller M Cloughesy T Portnow J . Isocitrate dehydrogenase (IDH) mutant gliomas: a Society for Neuro-Oncology (SNO) consensus review on diagnosis, management, and future directions. Neuro Oncol. (2023) 25:4–25. doi: 10.1093/neuonc/noac207 36239925 PMC9825337

[20] de la Fuente MI Touat M van den Bent MJ Preusser M Peters KB Young RJ . The role of vorasidenib in the treatment of isocitrate dehydrogenase-mutant glioma. Neuro Oncol. (2025) 27:1135–48. doi: 10.1093/neuonc/noae259 39723472 PMC12187374

[21] Kotecha R Schiff D Chakravarti A Fleming JL Brown PD Puduvalli VK . Multidisciplinary management of isocitrate dehydrogenase-mutated gliomas in a contemporary molecularly defined era. J Clin Oncol. (2024) 42:2588–98. doi: 10.1200/JCO.23.02195 38833641 PMC11283772

